# Human papillomavirus vaccine uptake and its determinants among women in Africa: an umbrella review

**DOI:** 10.3389/fpubh.2025.1537250

**Published:** 2025-06-05

**Authors:** Berihun Agegn Mengistie, Amlaku Nigusie Yirsaw, Gebeyehu Lakew, Gebrehiwot Berie Mekonnen, Adamu Ambachew Shibabaw, Alex Ayenew Chereka, Gemeda Wakgari Kitil, Wubet Tazeb Wondie, Alemken Eyayu Abuhay, Eyob Getachew

**Affiliations:** ^1^Department of General Midwifery, School of Midwifery, College of Medicine and Health Sciences, University of Gondar, Gondar, Ethiopia; ^2^Department of Health Promotion and Health Behavior, Institute of Public Health, College of Medicine and Health Sciences, University of Gondar, Gondar, Ethiopia; ^3^Department of Pediatrics and Child Health Nursing, College of Health Sciences, Debre Tabor University, Debre Tabor, Ethiopia; ^4^Department of Health Informatics, College of Health Science, Mattu University, Mattu, Ethiopia; ^5^Department of Midwifery, College of Health Science, Mattu University, Mattu, Ethiopia; ^6^Department of Pediatrics and Child Health Nursing, College of Health Sciences and Referral Hospital, Ambo University, Ambo, Ethiopia; ^7^University of Gondar Comprehensive Specialized Hospital, Gondar, Ethiopia

**Keywords:** Africa, human papillomavirus vaccine, HPV vaccine, umbrella review, uptake

## Abstract

**Background:**

Globally, cervical cancer is the fourth most prevalent disease among women. It is primarily caused by persistent infections with human papillomavirus (HPV). The World Health Organization (WHO) strongly recommends HPV vaccination for girls aged 9 to 14 years. Although HPV vaccination is the most effective form of primary prevention against cervical cancer, the accessibility and uptake of the HPV vaccine remain low in developing nations, particularly in Africa. Therefore, this umbrella review aimed to determine the pooled prevalence of human papillomavirus vaccine uptake and its determinant factors in Africa.

**Methods:**

The protocol was registered in the International Prospective Register of Systematic Reviews (PROSPERO) under reference number CRD42024560032. Eligible systematic review and meta-analysis (SRM) studies were retrieved from PubMed, Hinari, ScienceDirect, and Google Scholar. Data were extracted using Microsoft Excel 2019 and analyzed using Stata software (version 17). The methodological quality of the included studies was examined using A Measurement Tool to Assess systematic Reviews (AMSTAR 2). Publication bias was checked using a funnel plot and Egger’s test. A random-effects model (DerSimonian–Laird method) was used to estimate the pooled prevalence of HPV vaccine uptake. The I-squared (I^2^) test was performed to assess statistical heterogeneity among the included studies.

**Results:**

This umbrella review included five SRM studies conducted across Africa, encompassing a total of 707,005 study participants. The pooled prevalence of HPV vaccine uptake in Africa was 41.38% (95% CI: 34.70, 48.06). Women’s knowledge of HPV vaccination (AOR: 3.22, 95% CI: 1.64–6.33) and attitudes toward HPV immunization (AOR: 2.48, 95% CI: 2.18–2.81) were significantly associated with HPV vaccine uptake.

**Conclusion:**

The uptake of the HPV vaccine in Africa remains significantly lower (41.38%) than the WHO’s global HPV vaccination target of 90% by 2030. Therefore, increasing vaccine uptake requires promoting women’s knowledge and attitudes toward HPV vaccination through facility-based education and counseling, planned campaigns, community-based programs, and advocacy for HPV vaccination and cervical cancer prevention using various mass media platforms.

**Systematic review registration:**

Berihun Agegn Mengistie, Muluken Demeke, Abebaw Setegn. An Umbrella review of Human Papillomavirus (HPV) Vaccine Uptake and its predictors among females in Africa, 2024. PROSPERO 2024 Available from https://www.crd.york.ac.uk/PROSPERO/view/CRD42024560032.

## Introduction

Cervical cancer is the fourth most prevalent disease among women globally, with an estimated 604,000 new cases and 342,000 deaths reported across 185 countries in 2020 (1). In low-resource nations, such as sub-Saharan Africa (SSA), the disease has the highest prevalence (84%), and 88% of deaths are associated with it (2). In 2020, 1,00,000 women were infected with human papillomavirus (HPV) in Africa alone, with approximately 70,000 deaths, accounting for 21% of all cervical cancer-related deaths worldwide (3). In general, SSA bears the highest regional burden of HPV infection, accounting for an average of 24% of global cases (4). Over 99% of cervical cancer cases are caused by chronic infections with oncogenic or high-risk sexually transmitted strains of HPV (5, 6).

The HPV vaccine has been shown to be effective in preventing HPV-related malignancies (4). The inclusion of the HPV vaccine in the National Immunization Program (NIP) is expected to alter the course and burden of cervical cancer, particularly in the SSA region. Currently, approximately 50% of African nations offer HPV vaccination through the NIP (3). However, the implementation of HPV vaccination has not been as successful as expected, particularly for the second dosage, due to various challenges (7–10). In SSA countries where vaccination programs have been implemented, the average coverage for the final dose is only 20% (3). Since 2006, the United States Food and Drug Administration (FDA) has approved three HPV vaccines, all of which are recommended by the World Health Organization (WHO), including the bivalent vaccine (Cervarix), the quadrivalent vaccine (Gardasil), and the 9-valent vaccine (Gardasil 9) (11). The WHO and other researchers recommend a one- or two-dose HPV vaccine schedule for girls aged 9–14 years, a one- or two-dose schedule for girls and women aged 15–20 years, two doses with a 6-month gap for women over 21 years, and more than two doses for immunocompromised individuals (12, 13).

Each of the vaccines offers protection against the high-risk HPV strains 16 and 18; however, Gardasil provides extra protection for the 9–14 age group against HPV strains 6 and 11, and Gardasil 9 offers protection against HPV strains 6, 11, 31, 33, 45, 52, and 58 (14). These vaccines are most effective when administered before exposed to HPV; therefore, the WHO recommends vaccinating girls between the ages of 9 and 14 years (15, 16).

Worldwide, 39.7% of women are immunized against HPV, but this proportion is 68% in nations with high-income countries and 2.7% in low- and middle-income countries (LMICs) (17). A systematic review of the population-level effects of HPV vaccination over 58 years in 14 high-income countries found that the overall prevalence of HPV strains 16 and 18 decreased by 83% among girls aged 13–19 years and by 66% among women aged 20–24 years, resulting in substantial cross-protection against HPV strains 31, 33, and 45 (18). A single dose of the HPV vaccine provides comparable protection against highly susceptible HPV strains (types 16 and 18) as two or three doses of HPV vaccination (16). Cervical cancer is highly preventable and treatable if it is diagnosed at an early stage (19). The global implementation of the HPV vaccine, combined with early screening for cervical cancer, is expected to reduce the incidence of cervical carcinoma in the next decades. The WHO has launched a global initiative to scale up prevention, screening, and therapeutic efforts aimed at eradicating cervical carcinoma as a global health issue in the 21^st^ century (19).

In November 2020, the WHO introduced the 90–70-90 triple intervention approach, a global initiative aimed at eliminating cervical cancer as a public health concern. This global strategy aims to vaccinate 90% of girls against HPV by the age of 15 years, to screen 70% of women using a high-performance screening method twice between the ages of 35 and 45 years, and to treat at least 90% of women identified with precancerous cervical lesions or cervical cancer (19).

Over a decade of implementation programs has provided important lessons on raising vaccination coverage in various LMICs. However, compared to other parts of the globe, SSA has made only modest progress in the execution and success of national HPV vaccination programs, primarily due to challenges related to the accessibility and affordability of the vaccine (20).

Umbrella reviews involve the systematic collection and review of several systematic review and meta-analysis (SRM) studies conducted on a particular study topic. To date, several SRM studies have reported inconsistent prevalence rates of HPV vaccine uptake across Africa, ranging from 28.53 to 55% (21–25). Therefore, the aim of this umbrella review was to summarize the heterogeneous findings of existing SRM studies to generate conclusive findings regarding the coverage and determinants of HPV vaccine uptake in Africa. This comprehensive evidence will be valuable for healthcare professionals (HCPs), health managers, and policymakers in implementing evidence-based interventions to improve the uptake of the HPV vaccine in Africa. It is also considered a cost-effective primary preventive intervention for precancerous cervical lesions and cervical cancer.

## Materials and methods

### Study protocol

The protocol for this umbrella review was developed in compliance with the Preferred Reporting Items for Systematic Reviews and Meta-Analyses Protocols (PRISMA-P) statement. Initially, a search was conducted on PROSPERO to check for the presence of a similar umbrella review, and no such studies were found to have been registered. Then, the study’s protocol was established and registered (reference number: CRD42024560032).

### Searching strategy

Umbrella reviews involve the systematic collection and review of several SRM studies conducted on a particular study topic. This umbrella review aimed to systematically combine previous SRM studies on HPV vaccine uptake and its associated factors in Africa. It provides a more comprehensive and integrated understanding of the evidence regarding the HPV vaccine in Africa, allowing for the implementation of evidence-based strategies to scale up HPV vaccine uptake across the continent.

An inclusive literature search for SMR studies on HPV vaccine uptake and its associated factors in Africa was conducted using the Condition, Context, and Population (CoCoPop) framework across PubMed, Hinari, ScienceDirect, and Google Scholar. Publications were retrieved from a previous study that met the eligibility criteria. A search strategy was developed for the databases using a combination of keywords and Boolean operators (“AND” and “OR”). All systematic reviews and meta-analyses published between 1 January 2014 and 15 August 2024 were included. Furthermore, snowballing techniques were used to identify additional studies by reviewing the reference lists of the articles retrieved from the database searches. Finally, we used the following searching commands: systematic review and meta-analysis OR systematic reviews AND uptake OR use OR acceptance OR practice AND human papilloma virus vaccine OR human papillomavirus vaccination OR HPV vaccination OR HPV vaccine AND daughters OR females OR adolescent girls OR schoolgirls OR female students AND Africa OR “East Africa” OR “North Africa” OR “West Africa” OR “South Africa” OR “Sub-Saharan Africa” (Supplementary file 3).

### Inclusion and exclusion criteria

We included systematic reviews and meta-analyses of studies published in English that reported the prevalence and/or associated factors of HPV vaccine uptake in Africa. In addition, only studies published on and after 1 January 2014 were included in this umbrella review.

Articles were excluded for the following reasons: they did not measure the outcome of interest or they were narrative reviews, primary studies, qualitative reviews, expert opinions, case reports, editorials, correspondence, scoping reviews, or methodological studies.

### Study selection process

Based on the inclusion and exclusion criteria, all searched studies were imported into EndNote X8, a reference management software, to remove duplicate studies. After duplicates were removed, two reviewers (EG and BAM) independently assessed the titles and abstracts of the remaining articles to identify both potentially eligible articles and any articles for which eligibility could not be determined from the title and abstract alone. Then, the full texts of the remaining articles were examined to assess their eligibility according to the criteria.

### Measurement of the outcomes

The primary objective of this study was to determine the overall prevalence of HPV vaccine uptake in Africa. The prevalence was calculated by dividing the number of women who received the HPV vaccine by the total number of women in the study, then multiplying the result by 100. The second objective of this umbrella review was to identify the predictors of HPV vaccine uptake in Africa, which were evaluated using adjusted odds ratios (ORs) from previous SRM studies.

### Data extraction and management

Two authors (BAM and ANY) conducted data extraction from the included SRM studies using a standardized data abstraction form developed in an Excel spreadsheet. Articles were initially screened and selected based on their title and abstract, then the full text was reviewed. In cases of disagreement, discussions were held with additional reviewers to finalize the selection of articles to include in this umbrella review. Following the comprehensive search, potentially eligible publications were imported into EndNote. Duplicate studies were removed when two or more articles shared similar features. A structured data extraction process was designed and implemented using a Microsoft Excel spreadsheet.

For each SRM study, the following data were extracted: identification data (first author’s last name and publication year), prevalence of HPV vaccine uptake, factors associated with HPV vaccine uptake, odds ratios with 95% confidence intervals, the number of primary studies included in each SRM study, study area, total sample size, publication bias assessment methods, and risk of bias assessment methods and scores.

### Quality assessment

The quality of the included articles was evaluated by two separate reviewers using A Measurement Tool to Assess systematic reviews (AMSTAR 2). The new quality assessment tool builds upon the original AMSTAR. The specific quality of the included articles was assessed based on the 16 criteria of the AMSTAR 2 tool. The system categorizes the specific SMR evidence into four categories based on its quality: high, moderate, poor, and critically low. This umbrella review excluded articles with critically low-quality evidence (26) (https://amstar.ca/Amstar_Checklist.php) (Table 1).

**Table 1:** Methodological quality assessment of the included systematic review and meta-analysis studies using AMSTAR 2.

Authors	Q1	Q2	Q3	Q4	Q5	Q6	Q7	Q8	Q9	Q10	Q11	Q12	Q13	Q14	Q15	Q16	Score
Agimas et al. (21)	Y	Y	Y	Y	N	Y	Y	Y	Y	Y	Y	Y	Y	Y	Y	Y	15
Addisu et al. (22)	Y	Y	Y	Y	Y	Y	Y	Y	Y	Y	Y	Y	Y	Y	Y	Y	16
Asgedom et al. (23)	Y	Y	Y	Y	Y	Y	Y	Y	Y	Y	Y	Y	Y	Y	Y	Y	16
Zewdie et al. (24)	Y	PY	Y	Y	Y	Y	Y	Y	Y	Y	Y	Y	Y	Y	Y	Y	15
Derbie et al. (25)	Y	Y	Y	Y	Y	N	Y	Y	Y	Y	Y	Y	Y	Y	Y	Y	15

Y, yes; PY, partial yes; N, no.

### Data synthesis and statistical analysis

After the data were extracted using a Microsoft Excel spreadsheet, they were exported to the STATA 17 statistical software, where all statistical data analyses were carried out. The extracted data were displayed using texts, tables, and forest plots. The standard error of prevalence for each SRM study was analyzed using the binomial distribution. The combined prevalence of the SRM studies was checked for heterogeneity using the I-squared (I^2^) test. Heterogeneity among the included studies was classified as low, moderate, and high based on I-squared values of <25, 50–75%, and 75%, respectively (27).

A random-effects meta-analysis model with DerSimonian and Laird’s method was used to determine the overall estimate of HPV vaccine uptake in Africa. Subgroup analysis was performed by country, region, and study population to identify potential causes of heterogeneity among the studies. In addition, we conducted a leave-one-out sensitivity analysis to determine the effects of individual SRM studies on the pooled estimate. Then, forest plots and tables were used to display the pooled estimates for the continent, along with their corresponding 95% confidence intervals. Publication bias or small study effects were detected using funnel plot symmetry (27). In addition, the statistical significance of publication bias was assessed using Egger’s test, with a *p*-value less than 0.05 indicating the presence of publication bias (28). A trim-and-fill analysis was performed if significant publication bias was detected to correct for missing studies. Finally, the meta-analysis results were presented using forest plots, along with the odds ratio (OR) and a 95% confidence interval.

## Results

### Study selection

**Figure 1:**
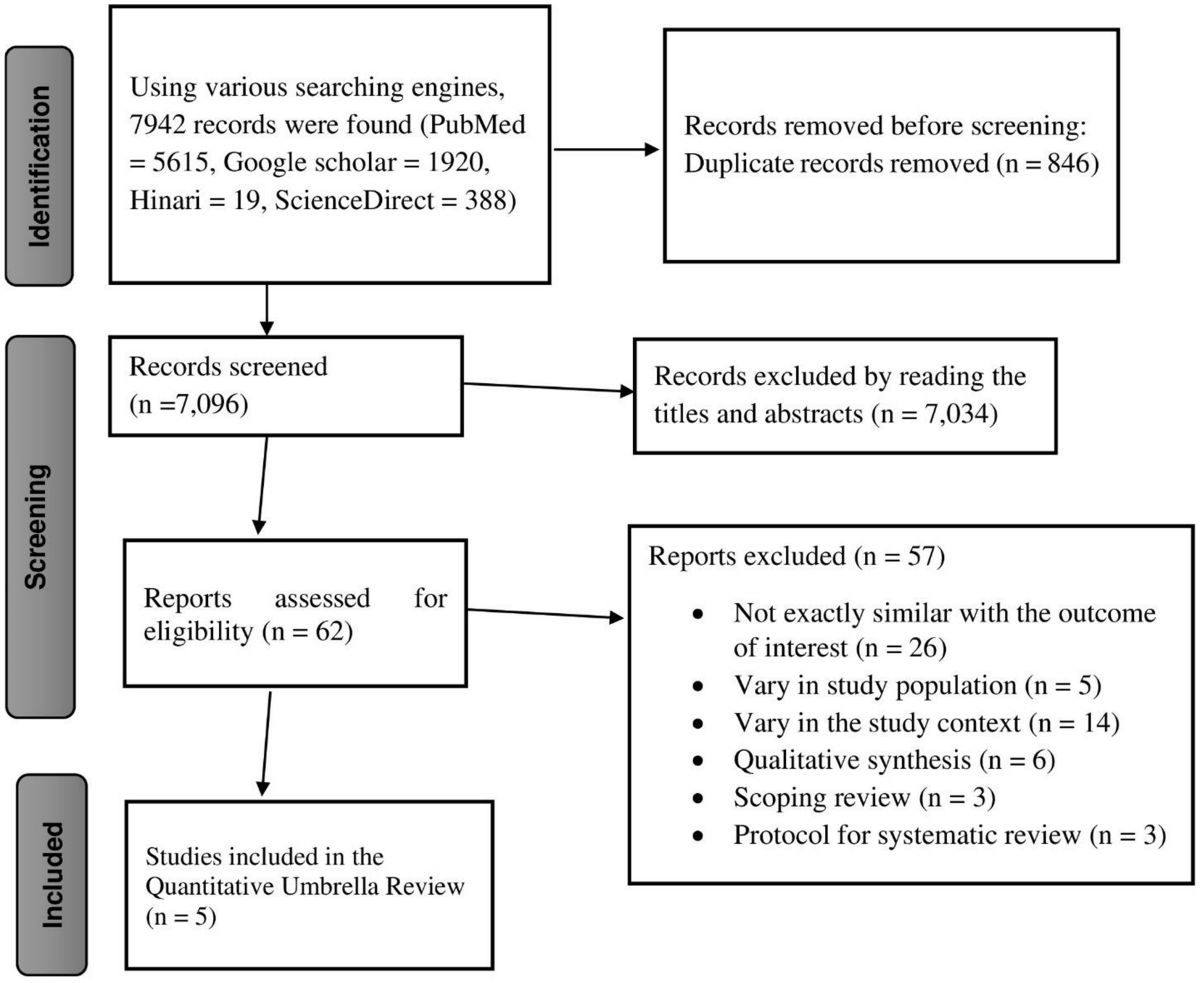
Prisma flow diagram showing the selection of studies for the umbrella review of HPV vaccine uptake in Africa.

A total of 7,942 studies were identified using four different search engines—PubMed, Hinari, ScienceDirect, and Google Scholar. Subsequently, 846 duplicate records were removed, leaving 7,096 studies eligible for screening. Of these, 7,034 articles were excluded based on title and abstract screening, resulting in 62 eligible studies. Among the 62 studies reviewed in full text, 57 were excluded for different reasons, including differences in study setting, irrelevance to the outcome of interest, and being qualitative systematic reviews, scoping reviews, or protocols for systematic reviews. Ultimately, five eligible studies were included in the final quantitative umbrella review (18, 22–25) (Figure 1).

### Characteristics of the included studies

In this umbrella review, five eligible SRM studies, comprising a total of 707,005 study participants, were included. In addition, there were 72 primary studies included in the eligible SRM studies. In terms of the distribution of the SRM studies across Africa, three studies were conducted in Ethiopia, one in East Africa, and the remaining study in SSA. Regarding the AMSTAR 2 quality score, the quality scores of each SRM article ranged from 15 to 16 (Table 2).

**Table 2:** Descriptive summary of the included systematic review and meta-analysis studies on HPV vaccine uptake among women in Africa.

Authors’ name	Study area	Study population	No. of_ studies	Sample size	HPV vaccine uptake (%)	AMSTAR score
Agimas et al. (21)	East Africa	All women	29	687,500	35	High
Addisu et al. (22)	Ethiopia	Adolescents	10	3,936	42.05	High
Asgedom et al. (23)	SSA	Adolescents	16	8,866	28.53	High
Zewdie et al. (24)	Ethiopia	Adolescents	7	3,315	46.52	High
Derbie et al. (25)	Ethiopia	Adolescents	10	3,388	55	High

### Uptake of the HPV vaccine in Africa

**Figure 2:**
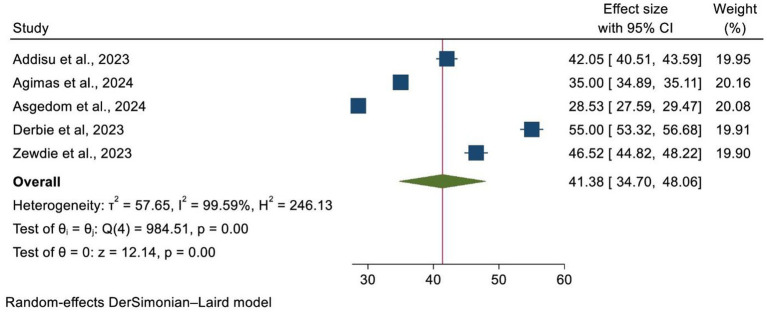
A forest plot that shows the pooled prevalence of HPV vaccine uptake in Africa.

In this umbrella review, the pooled prevalence of HPV vaccine uptake in Africa was found to be 41.38%, with a 95% confidence interval (34.70–48.06). The statistical test indicated the presence of significant heterogeneity among the included SRM studies (heterogeneity I^2^ = 99.59%, *p* = 0.00). As a result, a random-effects meta-analysis model was used (Figure 2).

### Publication bias

**Figure 3:**
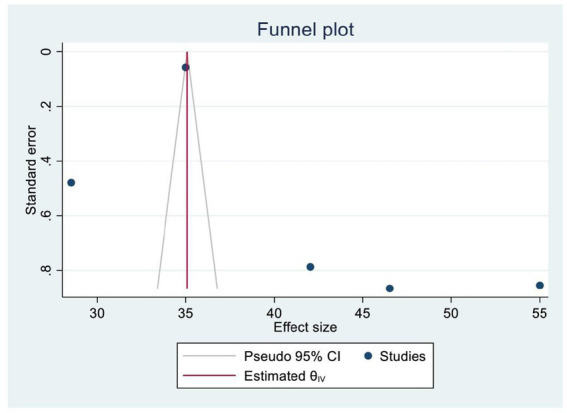
A funnel plot that shows the uptake of HPV vaccine in Africa.

A funnel plot and Egger’s test were used to assess publication bias. A symmetrical distribution in the funnel plot indicated the absence of publishing bias. In addition, we also examined publication bias statistically using Egger’s regression test, yielding a *p*-value of 0.086, which confirmed the absence of significant publication bias among the included SRM studies (Figure 3).

### Heterogeneity and subgroup analysis

**Figure 4:**
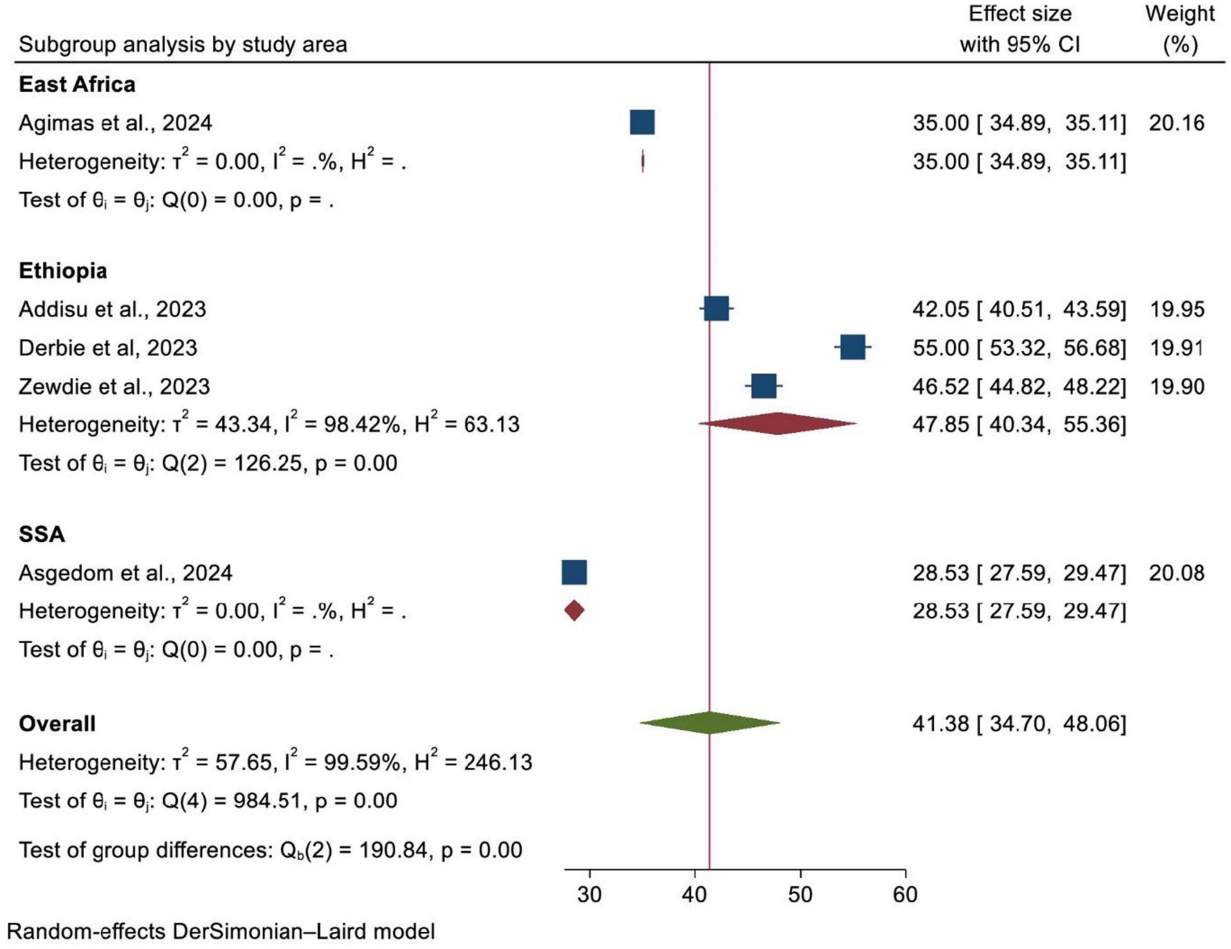
A forest plot showing the sub-group analysis of HPV vaccine uptake by study area.

**Figure 5:**
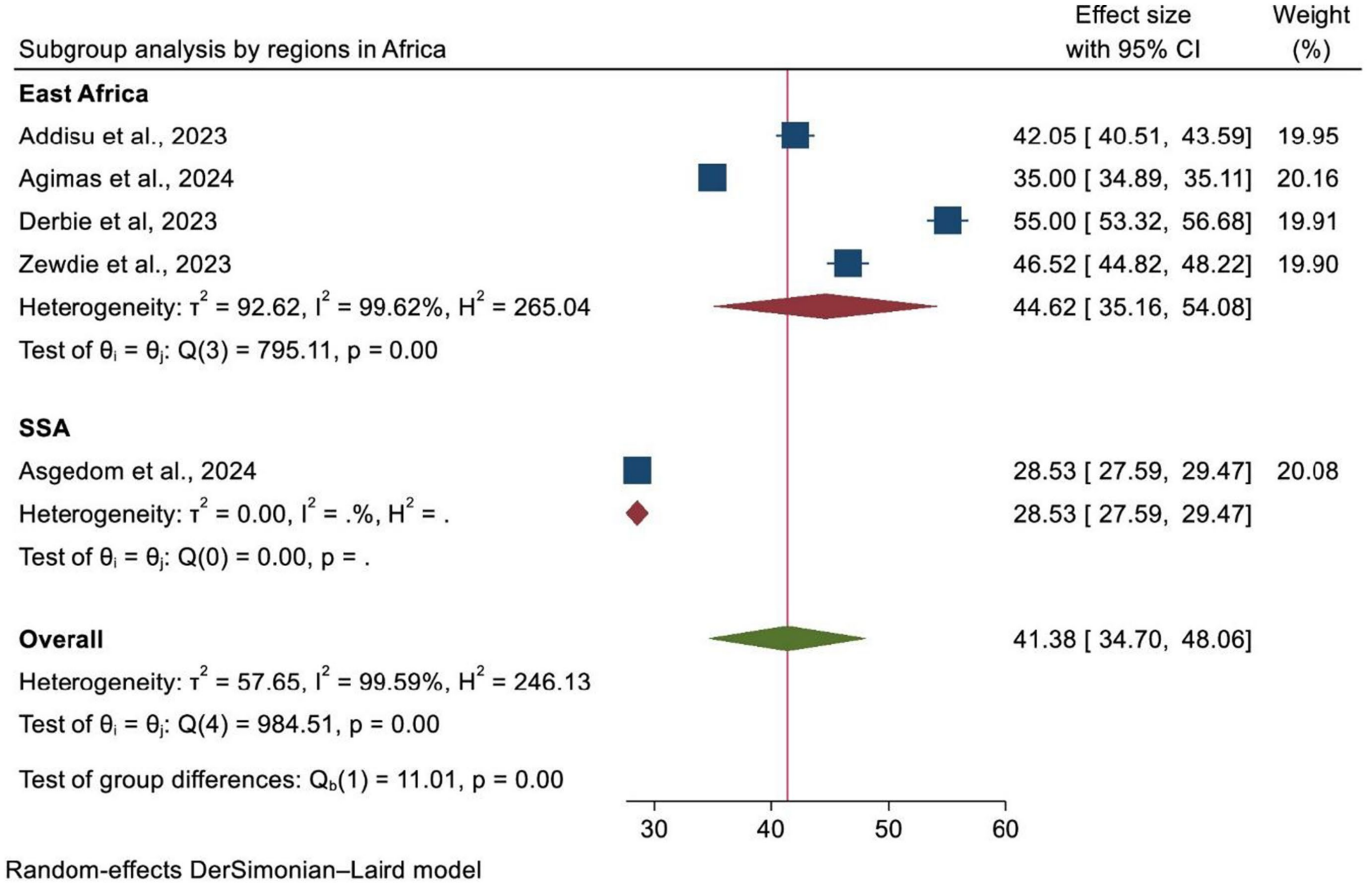
A forest plot showing the sub-group analysis of HPV vaccine uptake by region of Africa.

**Figure 6:**
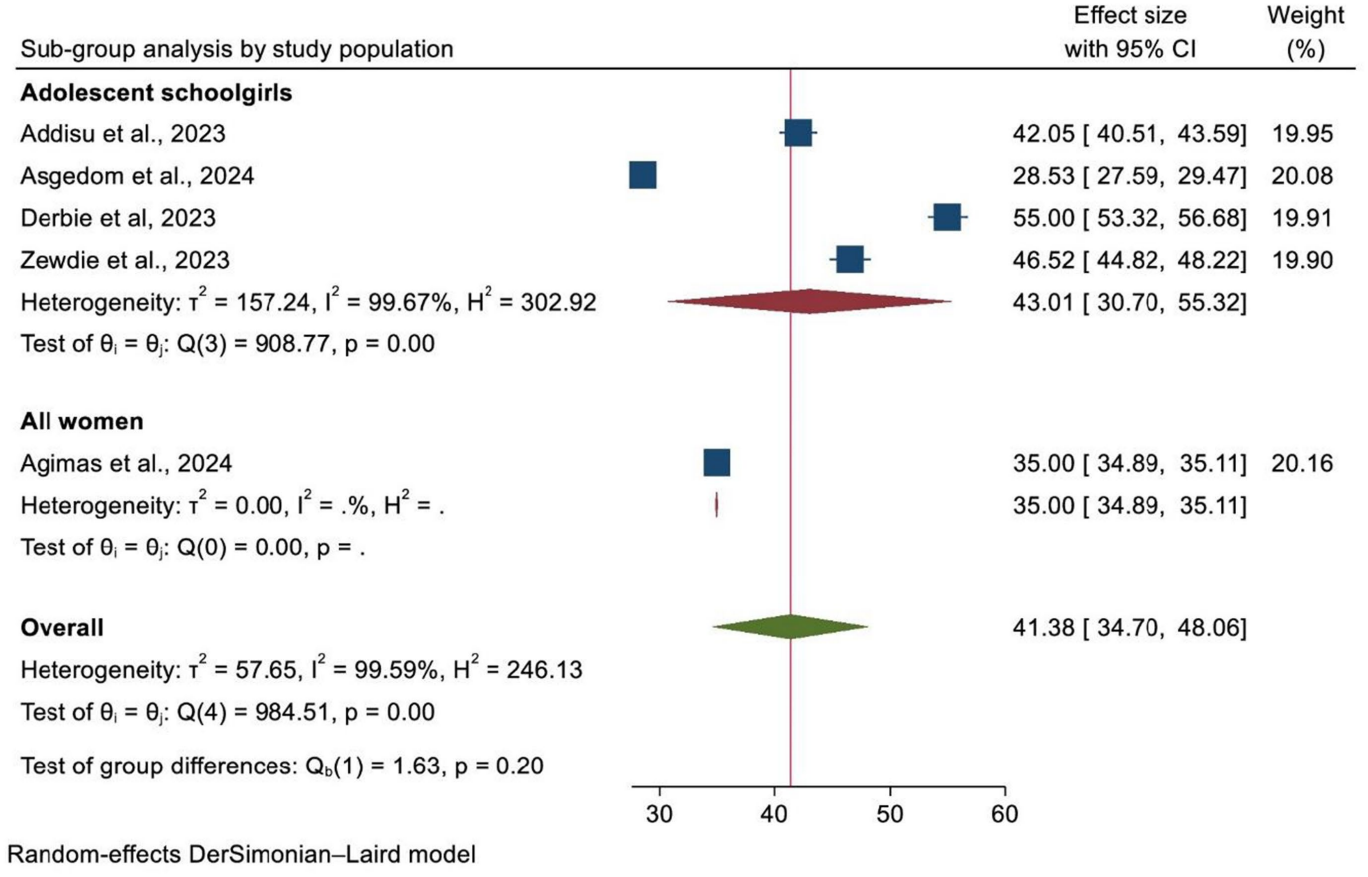
A forest plot showing the sub-group analysis of HPV vaccine uptake by the study population.

A subgroup analysis was carried out according to the country where the study was performed, the region of Africa, and the number of primary studies included in each SRM. As a result, Ethiopia and SSA had the highest and lowest overall prevalence of HPV vaccination at 47.85% (95% CI: 40.34–55.36) and 35.00% (95% CI: 34.89, 35.11), respectively (Figure 4). In addition, the highest prevalence of HPV vaccination was observed in East Africa at 44.62% (95% CI: 35.16–54.08). However, the lowest HPV vaccination coverage was observed in SSA, with a prevalence of 28.53% (95% CI: 27.59–29.47) (Figure 5). Lastly, a subgroup analysis based on the study population showed a higher pooled prevalence of HPV vaccination among adolescent schoolgirls, at 43% (95% CI: 30.70–55.32) (Figure 6). Despite performing subgroup analysis using the aforementioned criteria, there was no significant improvement in the variability of the overall prevalence of HPV vaccination.

### A leave-one-out sensitivity analysis

**Figure 7:**
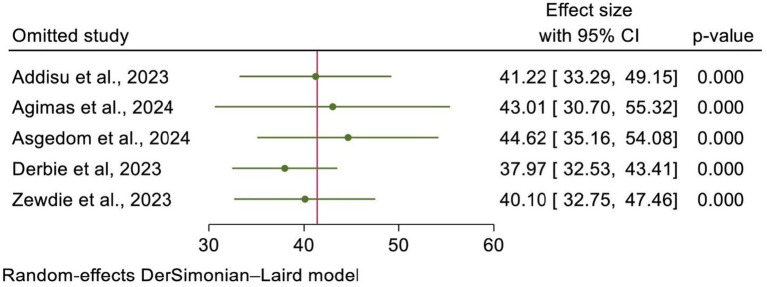
A one-leave-out analysis for uptake of HPV vaccine and its determinants in Africa.

Using the random-effects model, a leave-one-out sensitivity analysis was conducted to assess the effect of each individual SRM study on the effect size. The results indicated that no single study significantly influenced the pooled estimate, and the point estimate from its omitted analysis fell within the confidence interval of the combined analysis. This indicated the robustness of the overall estimate of HPV vaccine uptake in Africa (Figure 7).

### Meta-analysis of the factors associated with HPV vaccine uptake in Africa

**Figure 8:**
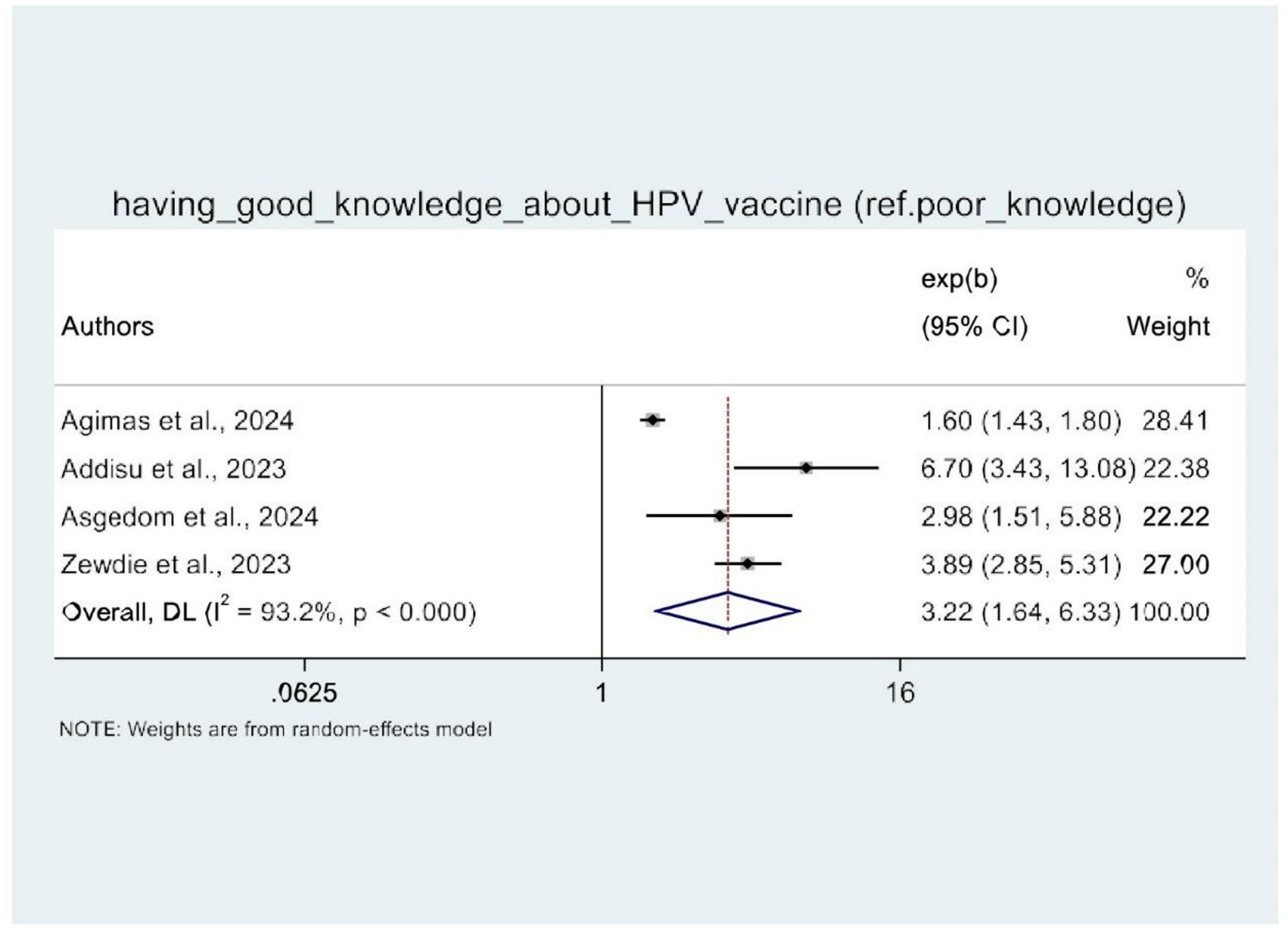
A forest plot that shows the pooled odds ratio of having good knowledge about HPV.

**Figure 9:**
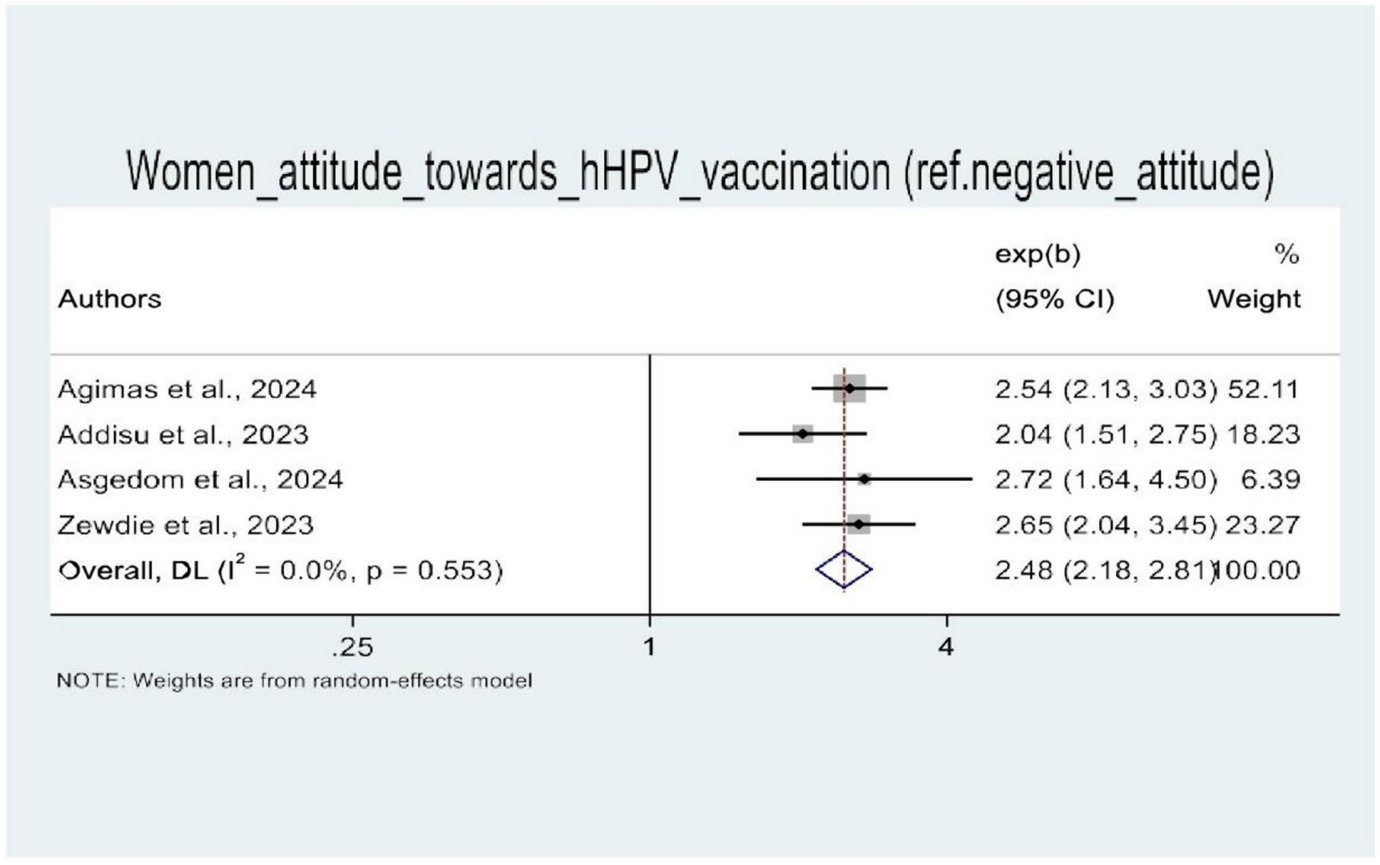
A forest plot that shows the pooled odds ratio of women’s attitude toward uptake of HPV vaccine in Africa.

In this umbrella review, four of the five SRM studies were examined for factors influencing HPV vaccine uptake (21–24). The random-effects model demonstrated that women’s knowledge of HPV vaccination and attitudes toward the HPV vaccine were significantly associated with HPV vaccine uptake. Therefore, four SRM studies showed the statistical significance of women’s knowledge about HPV vaccination. Women with good knowledge of the HPV vaccine (AOR: 3.22, 95% CI: 1.64–6.33) were three times more likely to be vaccinated against HPV as compared to their counterparts (Figure 8). Similarly, women with a positive attitude toward the HPV vaccine were three times more likely to receive the HPV vaccine (AOR: 2.48, 95% CI: 2.18–2.81) than those with a negative attitude toward the HPV vaccine (Figure 9).

## Discussion

Cervical cancer, caused by infection with HPV, is a significant threat to public health, particularly in low- and middle-income countries (LMICs) (9). However, it is a preventable type of cancer through the prevention of HPV infection (29). This can be accomplished via the highly effective and cost-efficient preventative technique employing the HPV vaccine, which protects against the most common strains of high-risk and low-risk HPV infections (30, 31). As of 2020, over 50% of WHO member nations had implemented HPV vaccination programs to support the 2030 Sustainable Development Goal (SDG) of reaching a 90% vaccination target (9). This umbrella review of systematic reviews and meta-analyses aimed to assess the pooled prevalence of HPV vaccination and its associated factors in Africa.

In this study, the pooled prevalence of HPV vaccination in Africa was 41.38% (95% CI: 34.70–48.06). This is in agreement with findings from China (32) and LMICs, where the prevalence was 38 and 45.48%, respectively (11). However, the finding of this umbrella review is much lower than the 90% target set by the WHO Global Strategic Plan for HPV vaccination by 2030 (18), the global estimate of 77% HPV vaccine coverage (9), and national estimates such as 66% in India (33), 62.8% in the USA (34), and 55.92% in Canada (35). This discrepancy might be due to differences in the accessibility of the HPV vaccine and disparities in socioeconomic status and healthcare systems across countries. The current study combined SRM studies conducted in African countries, which are classified as LMICs, while the remaining studies were conducted in developed nations with well-structured healthcare systems, extensive immunization programs, and higher levels of awareness among women regarding cervical cancer and HPV vaccination.

To achieve the WHO cervical cancer eradication goals, efforts must be made to boost HPV vaccine access and coverage in low- and middle-income countries (36). HPV immunization is a highly cost-effective form of primary prevention, and it has been commercially available for more than 10 years. However, the coverage of HPV vaccination remains low, and the burden of cervical cancer remains unacceptably high in Africa (11, 37). Adolescents who have not yet engaged in sexual activity can effectively prevent cervical cancer by receiving preventive HPV vaccines. However, in regions with limited resources, vaccine supply and accessibility continue to be significant challenges (38). As of 2023, 29 of 54 African countries have integrated HPV vaccination into their national immunization programs (38).

On this continent, vaccination program initiatives encounter a variety of institutional and individual barriers (39, 40). Challenges related to HPV vaccine inaccessibility and logistical problems include maintaining the cold chain, disruptions caused by the COVID-19 pandemic, delayed transportation, a shortage of healthcare professionals, and a lack of monitoring and evaluation (11, 41–43). Individual-level factors, such as vaccine hesitancy, poor uptake, and missing vaccinations for different reasons (financial issues, inaccessible health facilities, lack of awareness, socio-cultural factors, and misconceptions about HPV vaccination), collectively serve as barriers to HPV vaccination in the region (42, 44, 45). Furthermore, academic institutions are at the forefront of conducting investigations and producing important information required by policymakers and other stakeholders to guide the implementation of HPV vaccination programs (46, 47).

Furthermore, a subgroup analysis by study population also revealed that the uptake of the HPV vaccine was higher among adolescents (43.01%) as compared to other age groups. This might be associated with the fact that the majority of countries focus their efforts on vaccinating adolescent girls (9–14 years). It can also relate to the fact that countries have attempted to expand school-based immunization campaigns among adolescents, which might be a possible justification for the increase in HPV vaccination rates in this group.

In this study, it was found that women’s knowledge about the HPV vaccine and their attitude toward HPV vaccination are the two predictors that are significantly associated with the uptake of the vaccine. Accordingly, having good knowledge of cervical cancer and HPV vaccination is positively associated with increased HPV vaccine uptake. This finding is consistent with the findings of SRM studies in adolescents (48), in Italy (49), and in China (32, 50).

This could be explained by women’s strong understanding of cervical cancer and HPV vaccination, which enables them to make informed decisions, resolve concerns, and actively seek out the HPV vaccine, resulting in increased HPV vaccination coverage and improved public health.

In addition, women’s attitude toward HPV vaccine use was an important determining factor. Therefore, women with a good attitude toward the HPV vaccine were more likely to receive it than those with a negative attitude toward it. This pooled effect is consistent with findings from other systematic review and meta-analysis studies (48). The most plausible explanation for this is that a good attitude toward the HPV vaccine shows favorable views, reduced misconceptions, and greater motivation, all of which contribute to a higher likelihood of vaccine uptake among women.

Overcoming negative attitudes and fostering positive knowledge about the HPV vaccine is crucial for increasing vaccination rates and ultimately improving public health. Moreover, women with a positive attitude toward the vaccine are more likely to seek out information, understand the importance of vaccination, and make a well-informed decision to get vaccinated.

Finally, the findings of this umbrella review highlight that the current overall HPV vaccination coverage in Africa is far from meeting the WHO’s cervical cancer elimination strategy by 2030. Therefore, it is essential to advocate for and expand HPV vaccination programs in all countries, promoting collaborations and partnerships to make the HPV vaccine accessible, affordable, and available in low-income countries. In addition, encouraging community participation, implementing school-based campaigns, ensuring ongoing financial support, and, most importantly, conducting regular monitoring and evaluation of the program are some of the key interventions to scale up HPV vaccine uptake.

## Conclusion

In summary, the overall prevalence of HPV vaccine uptake among African women remains below half (41.38%) of the WHO’s target of 90% HPV vaccination coverage. Having good knowledge and a favorable attitude were found to be significantly associated with increased HPV vaccine uptake. Therefore, it is strongly recommended to promote awareness and understanding to increase knowledge about HPV vaccination and its benefits. In addition, addressing any unfavorable attitudes or misconceptions that may hinder the uptake of the HPV vaccine, as well as making the HPV vaccine more accessible and affordable for women in Africa, is crucial. This can be achieved through facility-based education and vaccination programs (school-based, institutional-based), planned campaigns, community-based advocacy, and the promotion of HPV vaccination and cervical cancer prevention measures via various mass media.

## Data Availability

The original contributions presented in the study are included in the article/Supplementary material, further inquiries can be directed to the corresponding author/s.
