# Supplementary material for: Human papillomavirus vaccine uptake and its determinants among women in Africa: an umbrella review
Source: Front Public Health. 2025 Jun 5;13:1537250. doi: 10.3389/fpubh.2025.1537250 (PMC12176891; doi:10.3389/fpubh.2025.1537250)
Supplement: Supplementary file 1 [file Supplementary_file_1.docx]

Searching strategies

**Google Scholar:** (Systematic review and meta-analysis OR Systematic reviews AND uptake OR use OR acceptance OR Practice AND Human papilloma virus vaccine OR Human papillomavirus vaccination OR HPV vaccination OR HPV vaccine AND daughters OR females OR adolescent girls OR school-girls OR female students AND Africa OR “East Africa OR “North Africa” OR “West Africa” OR "South Africa" OR “Sub-Saharan Africa”.

**PubMed:** (((((((((((((((Systematic review AND meta-analysis) AND (Systematic reviews) OR (uptake) OR (acceptance) OR (practice) OR (use) AND (Human papilloma virus vaccine) OR (Human papillomavirus vaccination) OR (HPV vaccination) OR (HPV vaccine) AND (daughters) OR (females) OR (adolescent girls) OR (school-girls) OR (female students) AND (Africa) OR (East Africa) OR (North Africa) OR (West Africa) OR (South Africa) OR (Sub-Saharan Africa))))))))))))))) Filtered in the last 10 years, Abstract, Free full article, Meta-analysis, Systematic Review

**ScienceDirect:** (Systematic review AND meta-analysis) AND (Human papilloma virus vaccine) OR (Human papillomavirus vaccination) AND (adolescent girls) OR (school-girls) AND Africa OR (Sub-Saharan Africa)

**Hinari:** (((((((((((((((Systematic review AND meta-analysis) AND (Systematic reviews) OR (uptake) OR (acceptance) OR (practice) OR (use) AND (Human papilloma virus vaccine) OR (Human papillomavirus vaccination) OR (HPV vaccination) OR (HPV vaccine) AND (daughters) OR (females) OR (adolescent girls) OR (school-girls) OR (female students) AND (Africa) OR (East Africa) OR (North Africa) OR (West Africa) OR (South Africa) OR (Sub-Saharan Africa)))))))))))))))
